# DNases improve effectiveness of antibiotic treatment in murine polymicrobial sepsis

**DOI:** 10.3389/fimmu.2023.1254838

**Published:** 2024-01-08

**Authors:** Jan-Fritjof Willemsen, Julia Wenskus, Moritz Lenz, Holger Rhode, Madgalena Trochimiuk, Birgit Appl, Laia Pagarol-Raluy, Daniela Börnigen, Corinna Bang, Konrad Reinshagen, Martin Herrmann, Julia Elrod, Michael Boettcher

**Affiliations:** ^1^ Department of Pediatric Surgery, University Medical Center Hamburg-Eppendorf, Hamburg, Germany; ^2^ Institute of Medical Microbiology, Virology and Hygiene, University Medical Center Hamburg-Eppendorf, Hamburg, Germany; ^3^ Institute of Clinical Molecular Biology, Kiel University, Kiel, Germany; ^4^ Department of Pediatric Surgery, University Hospital Mannheim, Heidelberg University, Mannheim, Germany; ^5^ Department of Medicine 3, Friedrich Alexander University Erlangen-Nuremberg and Universitäts-klinikum Erlangen, Erlangen, Germany; ^6^ Deutsches Zentrum Immuntherapie, Friedrich Alexander University Erlangen-Nuremberg and Universitätsklinikum Erlangen, Erlangen, Germany

**Keywords:** appendicitis, abdominal sepsis, biomarker, prospective, neutrophils, extracellular traps, NETs

## Abstract

**Introduction:**

Neutrophil extracellular traps (NETs) have various beneficial and detrimental effects in the body. It has been reported that some bacteria may evade the immune system when entangled in NETs. Thus, the aim of the current study was to evaluate the effects of a combined DNase and antibiotic therapy in a murine model of abdominal sepsis.

**Methods:**

C57BL/6 mice underwent a cecum-ligation-and-puncture procedure. We used wild-type and knockout mice with the same genetic background (PAD4-KO and DNase1-KO). Mice were treated with (I) antibiotics (Metronidazol/Cefuroxime), (II) DNAse1, or (III) with the combination of both; mock-treated mice served as controls. We employed a streak plate procedure and 16s-RNA analysis to evaluate bacterial translocation and quantified NETs formation by ELISA and immune fluorescence. Western blot and proteomics analysis were used to determine inflammation.

**Results:**

A total of n=73 mice were used. Mice that were genetically unable to produce extended NETs or were treated with DNases displayed superior survival and bacterial clearance and reduced inflammation. DNase1 treatment significantly improved clearance of Gram-negative bacteria and survival rates. Importantly, the combination of DNase1 and antibiotics reduced tissue damage, neutrophil activation, and NETs formation in the affected intestinal tissue.

**Conclusion:**

The combination of antibiotics with DNase1 ameliorates abdominal sepsis. Gram-negative bacteria are cleared better when NETs are cleaved by DNase1. Future studies on antibiotic therapy should be combined with anti-NETs therapies.

## Introduction

Neutrophils are classically regarded as the first line of defense of the innate immune system. They are essential in host defense against bacterial and fungal pathogens and protozoa. They are the most abundant immune cells in the human circulation. During acute infections, neutrophils are rapidly recruited to injured or infected areas and remain there for several days (1, 2). The short half-life of neutrophils in the circulation is balanced by a continuous and tightly controlled release from the bone marrow. In response to infection and injury, neutrophils form neutrophil extracellular traps (NETs)—high molecular weight chromatin filaments that serve as scaffolds decorated with histones and cytotoxic proteins, like myeloperoxidase (MPO) and neutrophil elastase (NE) (3, 4). NETs not only exhibit antimicrobial functions but also form during sterile inflammation (5–7). Excessive temporal and spatial production of NETs can have detrimental effects owing to their cytotoxic, pro-inflammatory, and prothrombotic activities. In fact, it has been shown that NETs contribute to the pathology of several inflammatory conditions, such as autoimmune diseases like systemic lupus erythematosus or rheumatoid arthritis, and ischemia reperfusion injury of intestine (volvulus) and testicle (testicular torsion) (5, 8–14). A common denominator of these disorders is the involvement of NETs as mediators of thrombosis and hyperinflammation and of the occlusion of vessels and ducts (13, 15–24).

Several studies have shown that overwhelming infections such as severe sepsis can lead to failure or paralysis of the immune system and that neutrophils may play an essential role in this process. Mechanistically, (I) release of incompetent or stunned neutrophils, (II) downregulation of their anti-microbial function, (III) neutrophil-mediated suppression of adaptive immunity, and (IV) neutrophil-mediated interference of microbe-associated molecular patterns (DAMPs) and danger-associated molecular patterns (MAMPs) seem to cause this paralysis and result in the inability to contain or eliminate infectious agents (25, 26). NETs trap microbes and immobilize them in areas with high concentrations of anti-microbial agents; some of these are released as components of NETs (3). DNA and histones are endowed with anti-microbial activities (27). However, excessive NETs and their degradation products can be detrimental in sepsis. The latter are prone to damage epithelia, endothelia, and various remote tissues including the liver and lung (28, 29).

Endothelial dysfunctions cause microcirculatory distress. In sepsis, this is the main cause of multiple organ failure. It causes tissue edema, disarrangement of hemostasis, and vasomotor control, and can eventually lead to death (30).

Some bacteria, such as pneumococci or meningococci, which have been associated with a severe clinical course of pneumonitis and meningitis, respectively, can evade NET-mediated killing (31). These bacterial strains remain trapped and protected within NETs without being killed by the immune system. In a rat model of meningitis, DNAse1, as NETs degrading treatment, improved clearance of *Streptococcus pneumoniae* and clinical outcomes.

It has been shown that the antimicrobial activity of NETs impairs the immune system’s efficacy to clear bacterial biofilm (32). Extracellular DNA released by immune cells reportedly act like an external secondary matrix, which shields the biofilm and enhances its resistance against antibiotic substances and phagocytosis (33). It is fair to assume that DNases facilitate phagocytosis by unmasking bacteria trapped in NETs. They were consequently accessible to intact neutrophils and antibiotics—comparable to the effects of DNases on biofilms. Hence, the aim of the current study was to evaluate the effect of the combination of antibiotic with anti-NETs therapy in cecal ligation and puncture (CLP), a polymicrobial murine model of severe abdominal sepsis.

## Methods

### Study design

C57BL/6 mice were utilized for the experimental model and were held within the animal facility, according to the German guide for the care and use of laboratory animals (Animal Welfare Act). The study was approved by the ethics committee of the Hamburg State Administration for animal research (N06/2020).

### Animal procedures

In the study, we made use of 6–8-week-old wild-type and PAD-4-knockout and DNase1-knockout mice; all mice had a similar genetic background. The animals were housed within the animal care facility receiving food and water *ad lib*. All animals were on the C57BL/6J background. *DNase1*-KO mice were generated as described earlier (5, 34, 35). WT and PAD4-KO mice were obtained from Jackson Laboratory. In order to induce abdominal sepsis, we performed a CLP (cecal ligation and puncture) procedure: (I) mice were anesthetized with 5% isoflurane gas (Forene 100%, AbbVie); (II) anesthesia was maintained with 2.5% isoflurane gas, administered via facemask; (III) after opening the abdominal cavity, the cecum was identified, ligated in its distal third and punctured with a 21G needle [as described earlier (36)]; and (IV) on day 4 after CLP all animals were euthanized after sedation with isoflurane gas.

To enhance standardization and avoid effects of varying gut microbiota, all CLP procedures were performed in siblings from the same litters co-housed before being separated into the treatment groups (37). Mice received their treatment 48 h after the CLP-procedure. Metronidazol (10 mg/kg bodyweight) and Cefuroxime (30 mg/kg bodyweight) were administered i.p. with a standardized volume of 0.1 ml in the corresponding groups. DNase1 was applied with a concentration of 10 mg/kg bodyweight. Treatment continued until euthanasia.

### Sample collection

After blood collection by cardiac punction, the animals were dissected using a midline incision, and the bowel was scrutinized, captured using a 4K/12-megapixel camera and finally prepped. The ligated cecum was aliquoted into test tubes containing either (1) Bouin solution or (2) liquid nitrogen. The latter was immediately stored at −80°C until further processing. Lung, liver, and intestine samples were collected into Eppendorf tubes. (1) One aliquot was frozen immediately in liquid nitrogen; (2) the second aliquot was incubated for 24 h in RNA-Later (Thermo-Fisher Scientific, Waltham, MA, USA, #AM7021) at 4°C and then stored at −80°C; and (3) the third aliquot was fixed in 4% formalin for 24 h, embedded in paraffin, and finally cut into 3-μm slices for staining. Blood samples were collected into EDTA tubes by cardiac punction, centrifuged at 2,000×*g* for 10 min at room temperature, and stored at −80°C.

### Histology

Upon tissue fixation in Bouin’s solution, intestinal tissue samples were dehydrated overnight, embedded in paraffin, and cut into 3-µm thick sections for further analysis. For quantifying intestinal mucosal tissue damage, the Chiu score was used, (38) and histopathological changes in lung and liver tissues were classified as none (0), mild (+1), moderate (+2), and severe (+3). To assess lung injury, we employed a modified version of the lung injury score system described by Engel et al.: (I) neutrophils in alveolar spaces, (II) neutrophils in interstitial space, (III) hyaline membranes, (IV) proteinaceous debris filling the airspaces, and (V) alveolar septal thickening (39). To assess liver injury, the scoring system described by Ito et al. was used, considering (I) areas of cell death, (II) degeneration (ballooning), and (III) inflammation around the central veins (40).

### Immunofluorescence

**Figure 1:**
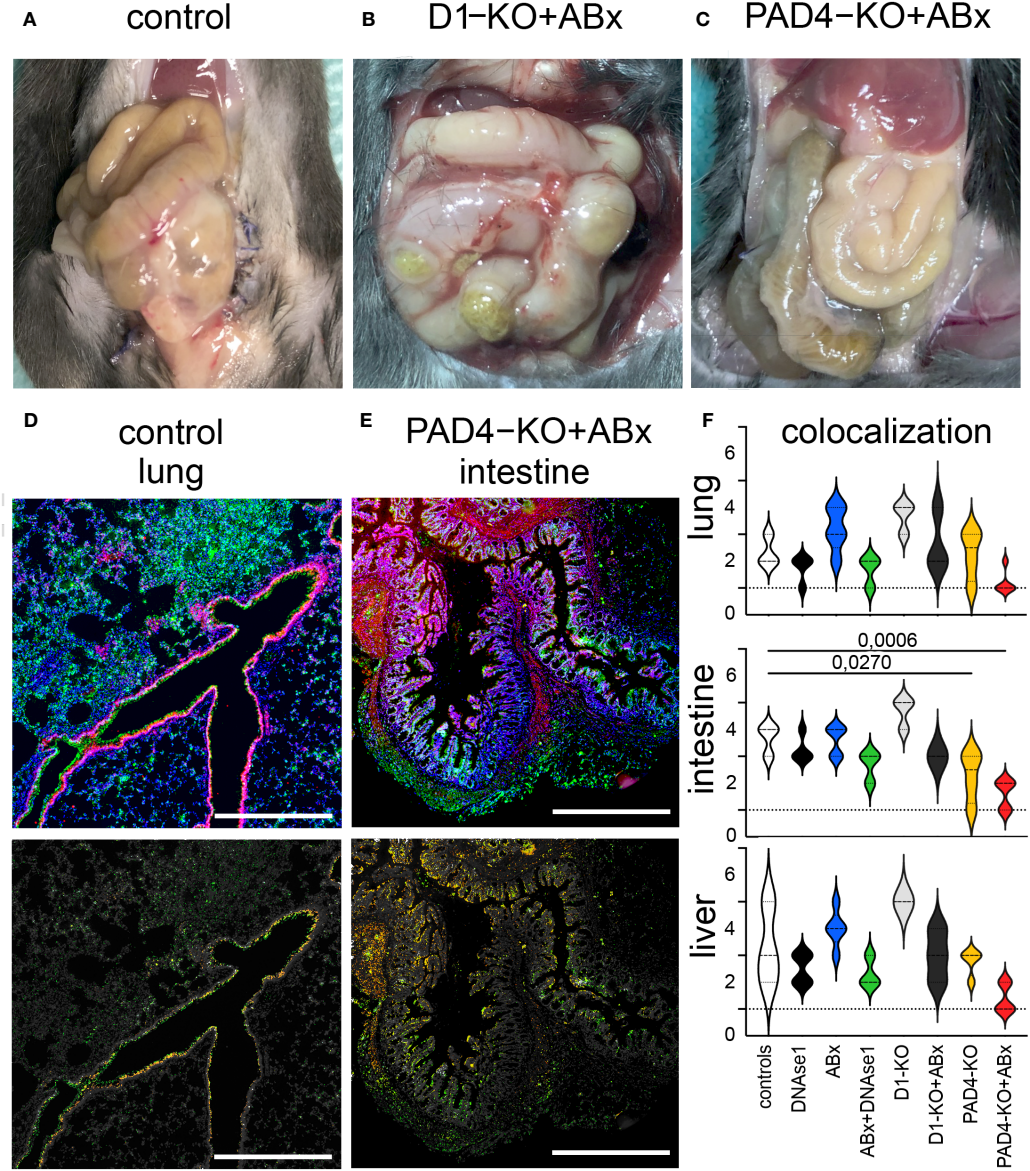
Macroscopic differences and colocalization of MPO/H3cit in immunofluorescence staining. **(A)** Open situs of a control animal. Note the swelling compared to **(C)**. **(B)** Open situs of a D1 knockout, treated with antibiotics. Note the vascular injections, adhesions, and swelling compared to **(C)**. **(C)** Open situs of a PAD4 knockout, treated with antibiotics. **(D)** Wide field image of IF staining of the lung, obtained from control animal (MPO green, H3cit red, DAPI blue), and assessment of colocalization (only places where both signals are present are colored). **(E)** Wide field image of IF staining of intestine, obtained from PAD4-KO treated with antibiotics (MPO green, H3cit red, and DAPI blue) and assessment of colocalization (only places where both signals are present are colored). **(F)** Evaluation of morphometry and colocalization of MPO/H3cit in the lung, intestine, and liver. Highest level of colocalization in the lung tissue was observed in wild-type mice treated with antibiotics. Significantly less colocalization was observed in the intestine of PAD4-mice treated with antibiotics compared to control animals. Highest level of colocalization in the intestine was observed in wild-type mice treated with antibiotics. Significantly less colocalization was observed in the liver tissue in PAD4-mice treated with antibiotics compared to control animals. Data shown as mean ± SD. Statistics: for comparison, one-way ANOVA with Dunnett´s correction.

Two different protocols for immune fluorescence were used: (I) for the evaluation of morphometry (**Figure 1F**) and (II) for the display of wide field images (**Figures 1D, E**) as described earlier (15, 41).

### Western blot

Before Western blot analysis, the tissues were homogenized using a Tissue Lyser LT (Qiagen, Hilden, Germany) at 50 Hz three times for 5 min each using cold Radioimmunoprecipitation assay buffer (RIPA) buffer (Sigma Aldrich, St. Louis, USA, #R0278), containing Complete Protease Inhibitor Cocktail 25× (Roche, Mannheim, Germany, #11697498001) and PhosStop Phosphatase Cocktail 10× (Roche, Mannheim, Germany, #04906845001). Steel beets were applied to crush the tissue. Bradford assays were performed utilizing a FlexStation 3 Multi-Mode Microplate Reader (Molecular Devices, San Jose, CA, USA) to determine the protein concentration of the lysates.

For Western blot analyses, we employed 1× Tris/glycine/sodium dodecyl sulfate (SDS) buffer (Bio-Rad, Hercules, CA, USA, #1610722) with proteins separated in a 8%–16% precast polyacrylamide gel electrophoresis (Bio-Rad, Hercules, CA, USA) and transferred to nitrocellulose membranes. Following the blocking with 5% bovine serum albumin or 5% delipidated milk (depending on the antibody), the membranes were incubated at 4°C overnight with specific primary antibodies (see supplement for antibodies) and GAPDH and Cyclophilin A as a loading control. The membranes were washed in TBS-T with 0.1% Tween (Serva, Heidelberg, Germany, #37470.01), incubated with a goat-anti-rabbit-IgG HRP antibody or a goat-anti-mouse-IgG Horseradish peroxidase (HRP) antibody (Antibodies Online, Aachen, Germany, #ABIN3020597, #ABIN3020588) for 1 h at room temperature. Finally, the blots were developed using a Medical Film Processor SRX-101A (Konica Minolta Medical & Graphic, Hino-Shi, Japan). The densitograms were analyzed using Image J software (Wayne Rasband, National Institute of Health, USA).

### Quantification of nucleosomes

NETs degradation products (particularly nucleosomes/histones) were quantified by a photometric sandwich enzyme immunoassay according to the instructions of the manufacturer (Roche Applied Science, Darmstadt, Germany (Cell Death Detection ELISA PLUS kit, #11920685001)). Mouse monoclonal antibodies directed against DNA and several histone antibodies served as catching and detecting antibodies, respectively.

### Microbiology

All blood, and tissue collection and homogenization were performed using aseptic techniques. Bacterial colonization was assessed using various types of agar plates (COS, MAC3, CAN). The tissue was homogenized using a Precellys Lysing Kit (Ref. no. P000918-LASYK0-A). Two dilutions of tissue homogenates (1:5; 1:100) from the different groups were analyzed for bacterial load. The quantification of colony-forming units was performed after 48 h incubation at 37°C. We also performed a 16s-rRNA-gene sequencing analysis in the Institute of Clinical Molecular Biology, Kiel University, using frozen native tissues from wild-type mice, treated with antibiotics, DNase1, or the combination of the two. Shannon Index was used as indicator for in-sample biodiversity.

### Proteomics

Liver samples were prepared and processed as described previously (42). We used an exploratory panel to determine the levels of 92 protein markers. Before analysis, the protein concentration of the liver lysates was determined and standardized. A proximity extension assay (PEA) combines the characteristics of an antibody-based immunoassay with polymerase chain reaction (PCR) technology. The principle of this sort of dual recognition immunoassay is that two matched antibodies, marked with DNA fragments, interact with different binding sites of a protein. This enables the DNA oligonucleotides to hybridize after getting close enough and consequently form a template, which is than amplified by PCR. This creates an antigen-specific DNA barcode. The concentration is proportional to the intensity of protein expression. The final analysis of the DNA barcodes is achieved using a microfluidic qPCR protocol (42).

### Statistics

We analyzed all data using SPSS Statistics 26 (IBM, NY, USA) and GraphPad Prism 9 (GraphPad, CA, USA) and performed a pre-power study calculation with G*Power 3.1. The power was deducted from a previous study examining necrotizing enterocolitis (NEC) and NETs in mice (2). We calculated survival analyses employing the Mantel–Cox test. We calculated the significance between groups with ANOVA and present the results as mean ± standard deviation (SD). For ordinal data, we calculated the significances with the Mann–Whitney test. The level of significance was set at <0.05.

## Results

**Figure 2:**
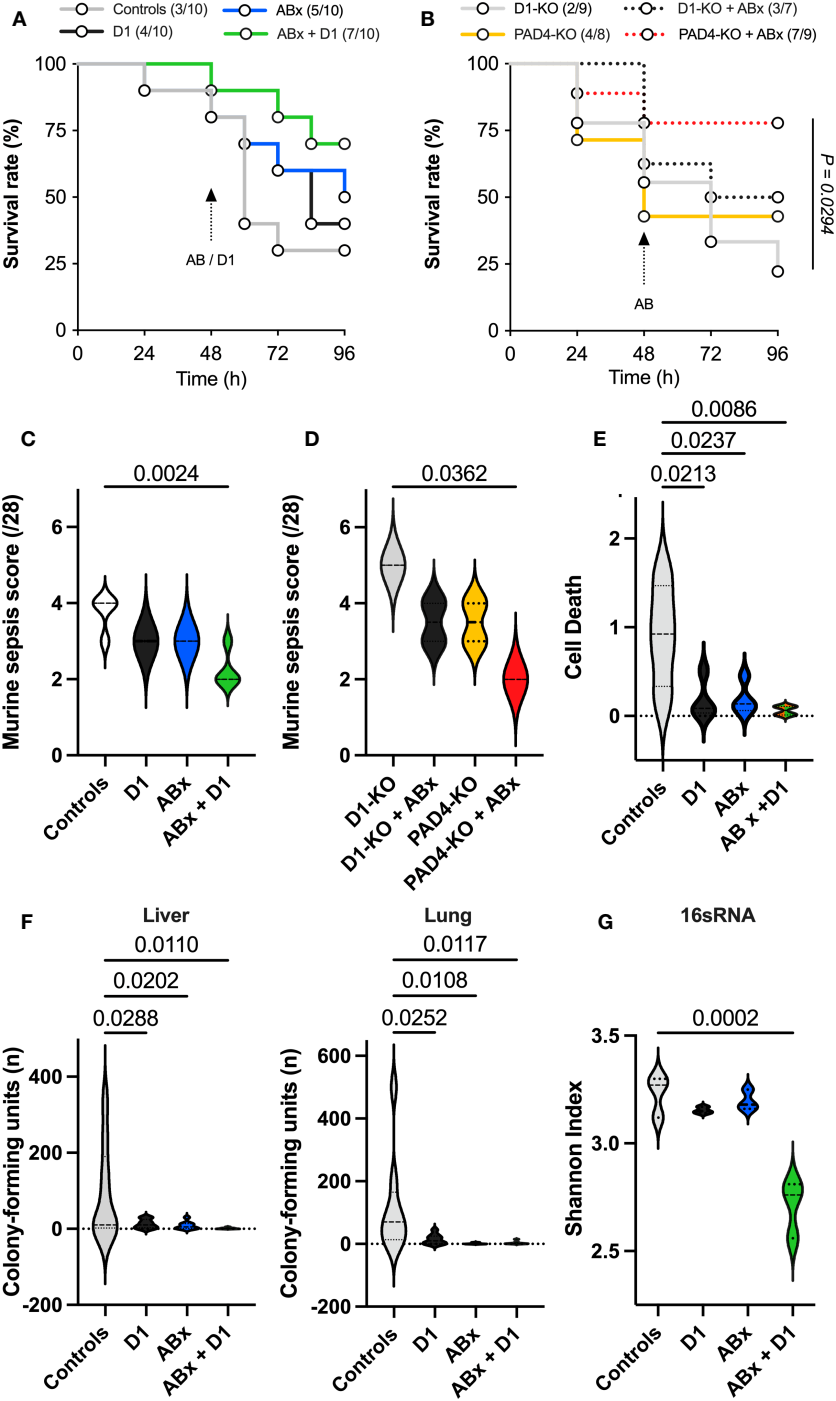
Assessment of survival, sepsis severity, and bacterial clearance. **(A)** Survival in wild-type mice, shown by the number of mice in the cohorts in the figure legend: treatment with D1 did not affect survival; however, antibiotic treatment improved the outcome. Mice treated with antibiotics and DNAse1 48 h after CLP procedure showed the best survival. Logrank test for trend showed no significant difference. **(B)** Survival in genetically modified mice, shown by the number of mice in the cohorts in the figure legend: PAD4-knockout mice treated with antibiotics showed significantly improved survival in the logrank test for trend (p=0,0294) compared to DNAse1-knockout mice. **(C)** Murine sepsis score in wild-type mice 96 h after CLP procedure: treatment only with D1 or antibiotics did not affect the outcome; however, combined treatment showed significantly lower sepsis score. **(D)** Murine sepsis score in genetically modified mice 96 h after CLP procedure: mice treated with antibiotics with reduced amount and size of NETs (PAD4-knockout) showed the lowest sepsis score compared to the other groups. **(E)** Assessment of nucleosomes: found significantly less NETs degradation products (nucleosomes/histones) in wild-type mice, when treated with D1 or antibiotics, particularly in mice, treated with the combination of antibiotics and D1. **(F)** Assessment of bacterial colonization in liver and lung tissues in wild-type mice: bacterial clearance was significantly improved in mice, treated with D1 or antibiotics. Best bacterial clearance was observed in mice treated with the combination of D1 and antibiotics. **(G)** Assessment of in-sample biodiversity via 16s-rRNA-gene sequencing analysis: bacterial biodiversity was significantly reduced in mice receiving the combination of D1 and antibiotics. Data shown as mean ± SD. Statistics: for comparison, one-way ANOVA with Dunnett’s correction or Kruskal–Wallis test with Dunn’s correction.

In total, 40 wild-type and 33 knockout mice (16 DNase1-KO, 17 PAD-4-KO) were utilized. Due to the severity of abdominal sepsis (sepsis scores; **Figures 2C, D**) induced by the CLP procedure (**Figure 2**), 35 animals survived until the end of animal procedures. After 72 h, 7/10 (70%) control animals had died; however, 7/10 PAD4 knockouts treated with antibiotics had survived (70%). Treatment with DNase1 alone did not affect the outcome, but antibiotic treatment improved survival. The best survival was observed in the wild-type group that received both DNase1 and antibiotics starting 48 h after the CLP procedure (**Figure 2A**). Additionally, the animals of this group had significantly lower sepsis scores (**Figure 2C**). This result was also reflected in knockout mice with reduced amount and size of NETs (PAD4-KO) when treated with antibiotics. This group showed significantly improved survival compared to DNase1 knockouts (**Figure 2B**). In this group, the lowest sepsis score in knockout animals was found (**Figure 2D**).

Treatment with DNase1 and/or antibiotics resulted in significantly reduced levels of histones and nucleosomes as surrogate markers for NETs degradation. This effect was most apparent in animals receiving the combination of DNase1 and antibiotics (**Figure 2E**).

**Figure 3:**
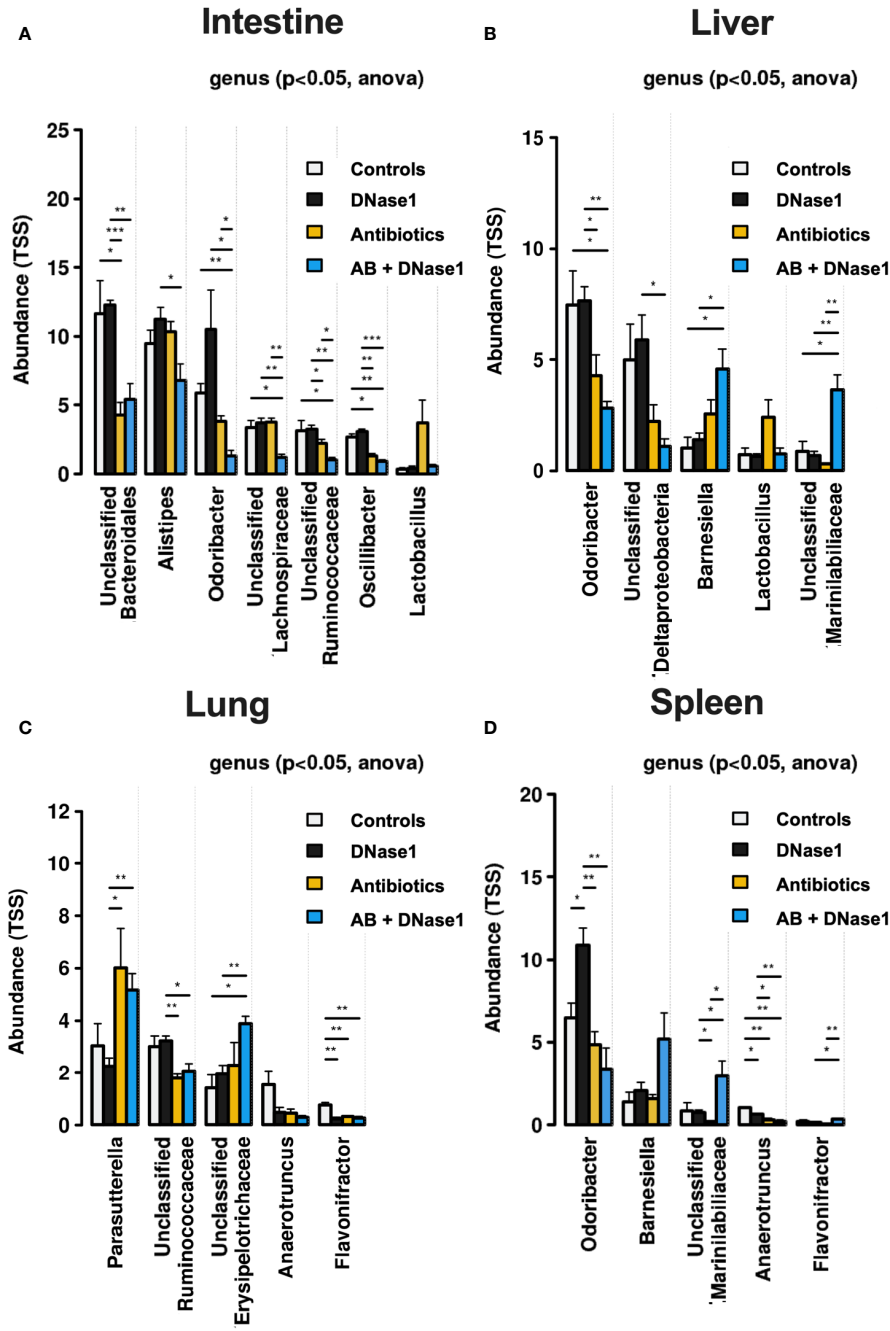
Spectral analysis of bacterial colonization in **(A)** intestine, **(B)** liver, **(C)** lung, and **(D)** Spleen. Not all bacteria seem to be equally affected by anti-NETs therapy. However Gram-negative bacteria are cleared significantly better when NETs are dissolved by D1 and treated with antibiotics. Data shown as mean ± SD. Statistics: for comparison, one-way ANOVA with Dunnett’s correction. *P ≤ 0.05, **P ≤ 0.01, ***P ≤ 0.001.

Treatment with DNase1 and/or antibiotics affected bacterial clearance in the liver and lung. As shown in **Figure 2F**, antibiotics significantly reduced the number of colony-forming units and bacterial diversity. The effect was most pronounced when antibiotics were combined with Dnase1. Spectral analysis showed that after combined Dnase1 and antibiotic treatment, Gram-negative bacteria were significantly reduced when compared with the control group (**Figure 3**). This results in a significantly reduced bacterial diversity as shown by 16s-RNA sequencing (**Figure 2G**). However, it appears that Dnase1 treatment alone may be detrimental. In the Dnase1-only group, Odoribacter was significantly elevated in the gut and spleen. Thus, not all bacteria are equally affected by anti-NETs therapy (**Figure 3**). Macroscopically distinct differences regarding swelling and/or vascular injections were observed after euthanasia (**Figures 1A–C**). Significantly less colocalization of MPO/H3cit was observed in morphometry of immune fluorescence in PAD4 knockouts treated with antibiotics compared to controls (**Figure 1F**).

In addition, we found increased levels of some proinflammatory marker proteins in mice without functional NETs in Western blot and proteome analyses (**Supplementary Figures 2**, **3**).

## Discussion

A recent study reported that some bacteria may evade neutrophil clearance when being trapped within NETs (31). We theorized that NETs have a biofilm-like function and that dissolution of NETs will free bacteria to make them more susceptible to antibiotics. Additionally, it has been proposed that DNases will release antimicrobials like cell-free DNA and NET-bound proteins, which will in turn intensify bacterial clearance (31). The current studies show that anti-NETs therapy indeed improves antibiotic therapy. The combined treatment or the reduced amount and size of NETs (PAD4-KO) (15) significantly reduced sepsis, inflammation, tissue damage, and mortality. The reason for this may be due to two factors: (1) reduced NETs-associated toxicity in the late stages of sepsis and (2) improved bacterial clearance as a consequence of dissolving the biofilm-like NET-structures and the release of the NET-bound proteins.

The degradation of NETs is mediated by two extracellular deoxyribonucleases (DNases). DNases can completely metabolize extracellular DNA including NETs. The most abundant extracellular deoxyribonucleases are members of the DNase1 protein family but differ in their origin and substrate affinity. DNase1 is expressed by non-hematopoietic tissues and preferentially cleaves protein-free DNA (43). DNases have anti-inflammatory effects either via (1) interaction of NETs and platelets or via (2) resolution of the self-amplifying loop between activated neutrophils and NETs (44). The effectiveness of DNase1 treatment in sepsis is complex, requiring critical timing for optimal efficacy. The impact of DNase treatment varies depending on the stage of infection; it can either promote the dissemination of pathogens or restrict excessive NET formation, thereby reducing inflammatory injury (45, 46).

Thus, the improved survival and tissue damage after anti-NETs treatment may not be a surprise. In previous studies, it was reported that PAD4-KO mice were partially protected from sepsis (37). This was often attributed to the prevention of hyperinflammatory, pro-thrombotic, cytotoxic effects of NETs in the late stages of sepsis (47). It appears that intense infections like sepsis and neutrophil activation results in a self-amplifying loop of activated neutrophils and NETs (48). Activated neutrophils produce NETs, and NETs itself activate neutrophils by oxidative stress or IL-1b/IL-18 (49, 50). Moreover, NETs influence other immune cells like macrophages. A recent study found that NETs activate macrophages to produce interferon I, which in turn activates neutrophils and induces NETs formation (51). The consequence is a vicious cycle, which may be stopped by dissolving NETs. DNases may free up bacteria that were entangled in the NETs. Thus, DNase treatment should be accompanied with antibiotics in the clinical setting.

One may assume that DNases have a specific effect on bacterial infection. Microbial analysis revealed that Gram-negative bacteria were significantly reduced in the current study, which may indicate that not all bacteria are equally affected by anti-NETs therapy in combination with antibiotics. The treatment combination in the current study appeared to affect particularly Gram-negative bacteria. Future studies should further examine which bacteria other than pneumococci or meningococci may be particularly capable of evading NET-mediated killing (31).

In the current study, anti-NETs therapy did not significantly mitigate lung injury in the CLP model, which contradicts some previous studies (52–54). However, it has been established that various factors affect the relatively robust CLP model like number of cecal punctures, needle size, bacterial colonization, genetic background, sex, and age (55–57). Moreover, in recent studies, only minor signs of lung injury have been observed after CLP (58, 59), thereby challenging the notion of universal CLP-associated lung injury.

Recently, PAD4 inhibitors have been evaluated as a treatment option to reduce NET formation. Citrullination of histones may be an essential step in the pathology of polymicrobial sepsis (60). Histones released into the extracellular space play a significant role in contributing to endothelial dysfunction, organ failure, and mortality (10). It appears that PAD4 drives immune-mediated diseases and inflammatory disorders. Thus, PAD4 inhibitors may be advantageous by preventing NET formation compared to dissolving NETs using DNase1 (61). A head-to-head comparison for both treatment strategies is warranted in future studies.

In conclusion, it appears that the combination of antibiotics with anti-NETs therapy using DNases is very beneficial in the context of (abdominal) sepsis. Gram-negative bacteria appear to be cleared far better when NETs are dissolved. Our findings appear to bridge the gap between reports of mice lacking NETs showing improved survival in various sepsis models. Future studies should further evaluate if antibiotic therapy should always be combined with anti-NETs therapy.

## Data availability statement

The original contributions presented in the study are publicly available. This data can be found here: [Proteomics Data: https://zenodo.org/records/10156238] [16s-RNA-Analysis: NCBI accession: PRJNA1042954].

## Ethics statement

The animal study was approved by Hamburg State Administration for animal research (N06/2020). The study was conducted in accordance with the local legislation and institutional requirements.

## Author contributions

JW: Validation, Writing – original draft, Investigation, Project administration, Visualization. JW: Investigation, Project administration, Validation, Conceptualization, Data curation, Formal Analysis, Writing – review & editing. ML: Data curation, Investigation, Validation, Writing – review & editing, Supervision. HR: Investigation, Supervision, Validation, Writing – review & editing, Conceptualization, Methodology. BA: Investigation, Methodology, Supervision, Validation, Writing – review & editing. MT: Data curation, Investigation, Methodology, Writing – review & editing. LP: Investigation, Supervision, Validation, Writing – review & editing, Conceptualization. DB: Investigation, Supervision, Validation, Writing – review & editing, Data curation, Methodology. CB: Investigation, Methodology, Supervision, Validation, Writing – review & editing, Software. KR: Investigation, Methodology, Supervision, Validation, Writing – review & editing, Conceptualization, Resources, Visualization. MH: Methodology, Supervision, Validation, Visualization, Writing – review & editing, Data curation. JE: Data curation, Methodology, Supervision, Validation, Visualization, Writing – review & editing, Investigation, Project administration, Software. MB: Data curation, Methodology, Supervision, Validation, Writing – review & editing, Conceptualization, Formal Analysis, Funding acquisition, Resources, Writing – original draft.

## Supplementary material

Supplementary Figure 1 Differences in immunohistochemistry. (HE-staining/Ly6G-staining). **(A)** Intestine: No significant differences were observed, neither in Chiu-score, nor in Ly6G- staining. **(B)** Liver: No significant differences were observed, neither in HE-, nor in Ly6G-staining. **(C)** Lung: Significant differences between PAD4-knockouts treated with antibiotics and controls were observed in Ly6G-staining. PAD4-knockouts treated with antibiotics showed the highest score in HE-staining as well (no significant difference). Data shown as mean ± SD. Statistics: For comparison, one-way ANOVA with Dunnett´s correction.

Supplementary Figure 2 Western blot analysis of various inflammation associated proteins in liver and lung tissue. **(A)** TNF alpha: No inter group differences were observed in liver tissue. However, we significantly elevated levels of TNF alpha were found in the PAD4-knockout group treated with antibiotics compared to controls in lung tissue. **(B)** Interleukin 1ß: Significant differences between wildtype mice treated with antibiotics, PAD4-knockouts and even PAD4-knockouts treated with antibiotics were observed in liver tissue. Also, in lung tissue significant inter group differences compared to control animals were observed. **(C)** Nuclear factor kappa B p65: No significant inter group differences could be found in liver tissue. However, highest values were measured in wildtype mice treated with antibiotics. In lung tissue significant inter group differences in comparison to the control group were found. **(D)** Interleukin 10: No significant inter group differences were observed in liver tissue. In lung tissue wildtype mice treated with D1 and D1-knockouts showed significantly elevated levels. **(E)** Cxcr1: Significant differences between controls and D1-knockout treated with antibiotics were observed in liver tissue. In lung tissue, significantly elevated levels were observed in wildtype mice treated with D1 compared to controls. Data shown as mean ± SD. Statistics: For comparison, one-way ANOVA with Dunnett´s correction.

Supplementary Figure 3 Proteomic analysis of various inflammation associated proteins in liver tissue. No significant inter group differences were found. Data shown as mean ± SD. Statistics: For comparison, one-way ANOVA with Dunnett´s correction.
