# Supplementary material for: DNases improve effectiveness of antibiotic treatment in murine polymicrobial sepsis
Source: Front Immunol. 2024 Jan 8;14:1254838. doi: 10.3389/fimmu.2023.1254838 (PMC10801052; doi:10.3389/fimmu.2023.1254838)
Supplement: Supplementary Figure 1 — Differences in immunohistochemistry. (HE-staining/Ly6G-staining). (A) Intestine: No significant differences were observed, neither in Chiu-score, nor in Ly6G- staining. (B) Liver: No significant differences were observed, neither in HE-, nor in Ly6G-staining. (C) Lung: Significant differences between PAD4-knockouts treated with antibiotics and controls were observed in Ly6G-staining. PAD4-knockouts treated with antibiotics showed the highest score in HE-staining as well (no significant difference). Data shown as mean ± SD. Statistics: For comparison, one-way ANOVA with Dunnett´s correction. [file DataSheet_1.pdf]

Supplementary Material:

Supplement 1: Differences in immunohistochemistry. (HE-staining/Ly6G-staining)

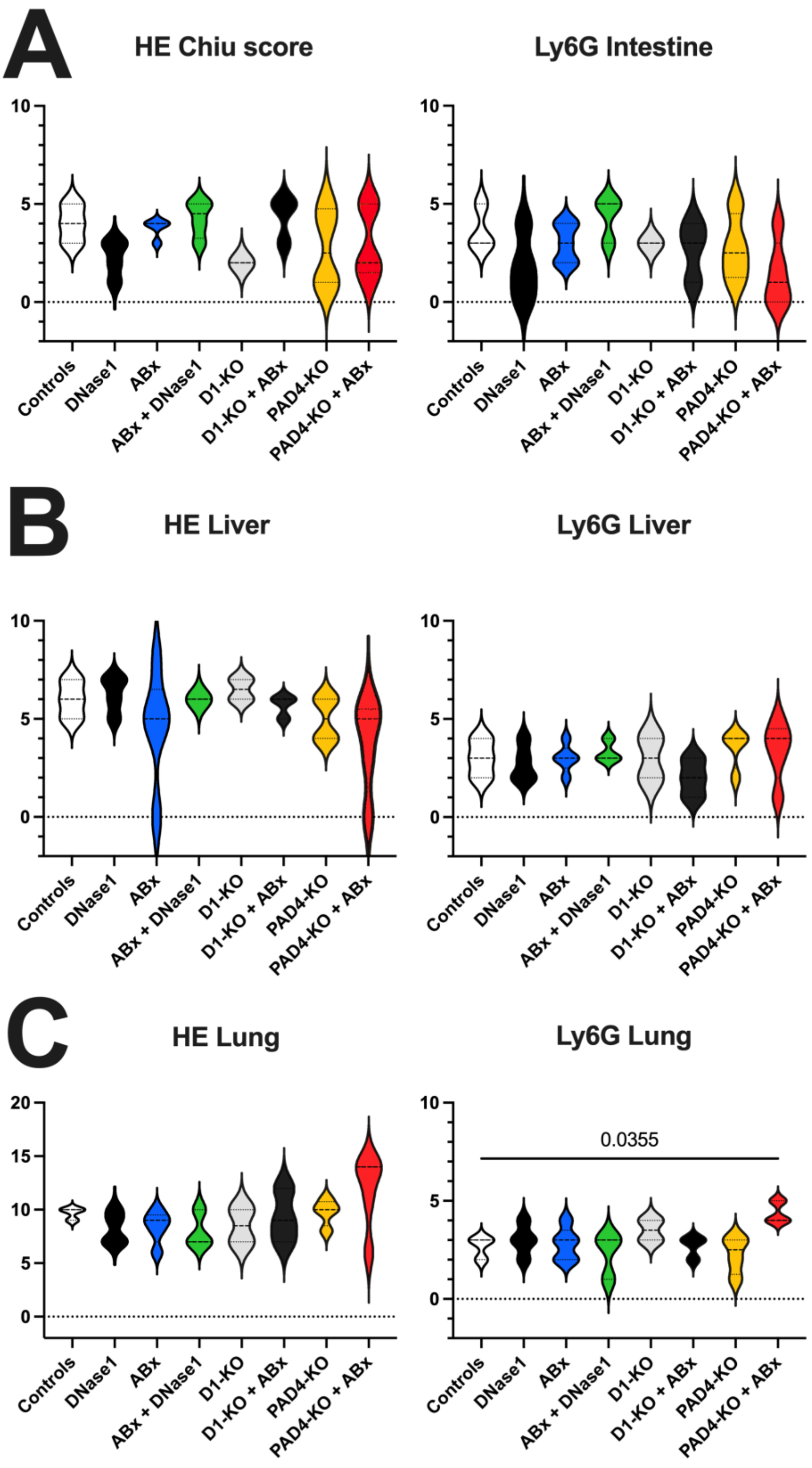

Supplement 2: Western blot analysis of various inflammation associated proteins in liver and lung tissue

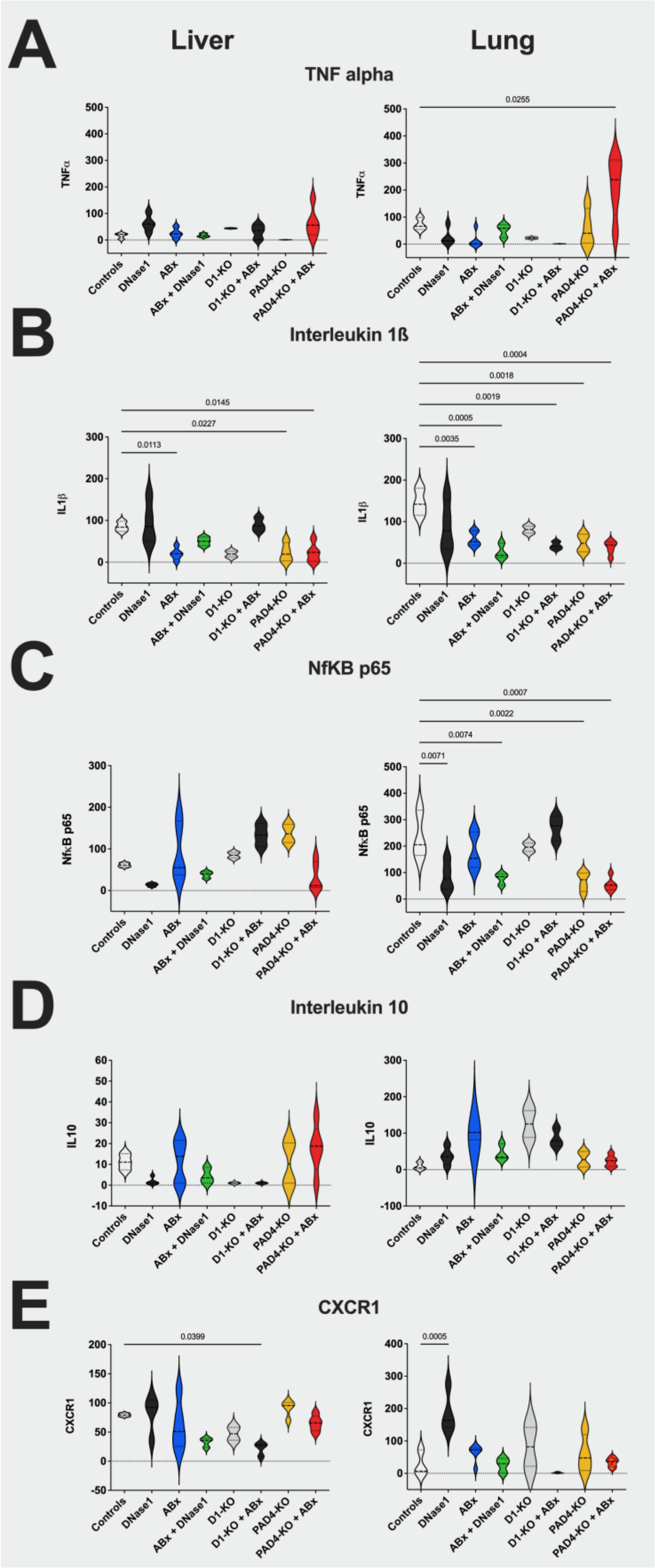

Supplement 3: Proteomic analysis of various inflammation associated proteins in liver tissue

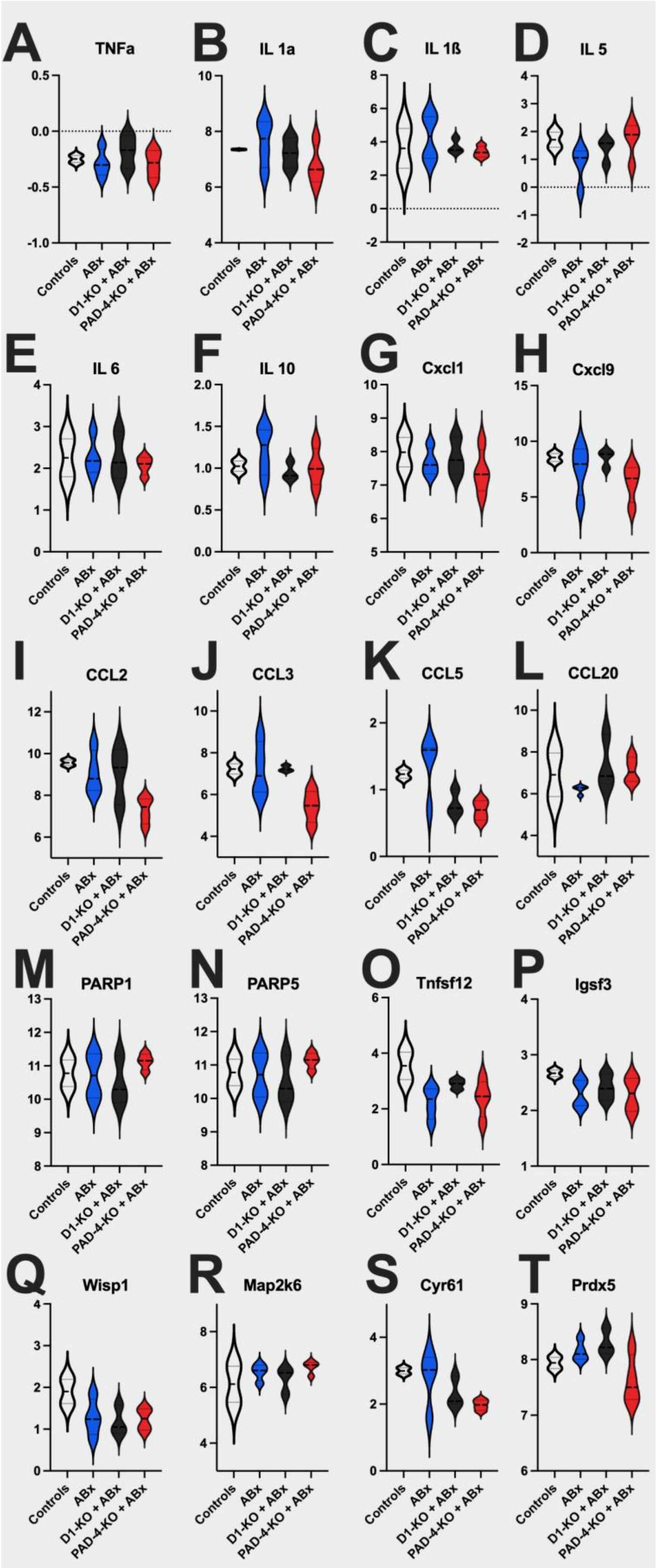

Antibody Table:

Western Blot: Primary Antibodies

Antibody	Producer	Dilution
**Tumor Necrosis Factor α**	Thermo-Fisher Scientific<br>(Waltham, Massachussetts, USA)<br>#PA5-19810	1:500
**Interleukin 1ß**	Thermo-Fisher Scientific<br>(Waltham, Massachussetts, USA)<br>#MM425B	1:3000
**Interleukin 10**	Thermo-Fisher Scientific<br>(Waltham, Massachussetts, USA)<br>#MM010	1:1000
**Nuclear-Factor kB-p65**	Abcam<br>(Cambridge, UK)<br>#ab16502	1:5000
**CXCR1**	Proteintech<br>(Rosemont, Illinois, USA)<br>#55450-1-AP	1:1000
**GAPDH<br>(Loading control)**	Abcam<br>(Cambridge, UK)<br>#ab181602	1:20000
**Cyclophilin A<br>(Loading control)**	Cell Signaling<br>(Danvers, Massachusetts, USA)<br>#2175S	1:10000

Western Blot: Secondary Antibodies

Secondary Antibody	Producer	Dilution
**Goat-anti-Rabbit<br>HRP**	Antibodies online<br>(Aachen, Germany)<br>ABIN3020597	1:10000
**Goat-anti-Mouse<br>HRP**	Antibodies online<br>(Aachen, Germany)<br>ABIN3020588	1:10000

IF (I): Primary Antibodies

Antibody	Producer	Dilution
**Neutrophil Elastase (NE)**<br>**Rabbit polyclonal**	Abcam<br>(Cambridge, UK)<br>#ab68672	1:100
**NE iso control**<br>**Rabbit polyclonal**	Abcam<br>(Cambridge, UK)<br>#ab37415	1:1000
**Histone H3cit (R8-Clone)**<br>**Rabbit monoclonal**	Abcam<br>(Cambridge, UK)<br>#ab219406	1:100
**H3-cit iso control**<br>**Rabbit monoclonal**	Abcam<br>(Cambridge, UK)<br>#ab172730	1:300
**Myeloperoxidase (MPO)**<br>**Goat polyclonal**	R&D Systems<br>(Minneapolis, Minnesota, USA)<br>#AF3667	1:20
**MPO iso control**<br>**Goat polyclonal**	R&D Systems<br>(Minneapolis, Minnesota, USA)<br>#AB-108-C	1:100

IF (I): Secondary Antibodies

Antibody	Producer	Color	Dilution
**NE donkey anti rabbit**	Jackson Immunoresearch<br>Philadelphia, Pennsylvania, USA<br>#711-605-152	AF 647	1:200
**MPO donkey anti goat**	Jackson Immunoresearch<br>Philadelphia, Pennsylvania, USA<br>#705-165-147	Cy3	1:200
**H3cit donkey anti goat**	Jackson Immunoresearch<br>Philadelphia, Pennsylvania, USA<br>#711-605-152	AF 647	1:200

IF (II): Primary Antibodies

Antibody	Producer	Dilution
**Histone H3cit (R2, 8, 17-Clone) Rabbit polyclonal**	Abcam (Cambridge, UK) #ab281584	1:300
**Myeloperoxidase (MPO) Rabbit polyclonal**	Abcam (Cambridge, UK) #ab9535	1:200
**Neutrophil Elastase (NE) Goat polyclonal**	Santa Cruz (Dallas, Texas, USA) #Sc-9521	1:100

IF (II): Secondary Antibodies

Antibody	Producer	Color	Dilution
**IgG goat anti rabbit**	Jackson Immunoresearch Philadelphia, Pennsylvania, USA #111-175-144	Cy5	1:400
**IgG goat anti rabbit**	Abcam (Cambridge, UK) #ab175471	AF 568	1:400
**IgG donkey anti goat**	Jackson Immunoresearch Philadelphia, Pennsylvania, USA #705-175-147	Cy5	1:400
